# Fibrin-coated collagen fleece versus absorbable dural sealant for sellar closure after transsphenoidal pituitary surgery: a comparative study

**DOI:** 10.1038/s41598-022-12059-x

**Published:** 2022-05-14

**Authors:** Julien Spitaels, Justin Moore, Nathalie Zaidman, Isabel Fernandes Arroteia, Geoffrey Appelboom, Sami Barrit, Sébastien Carlot, Viviane De Maertelaer, Sergio Hassid, Olivier De Witte

**Affiliations:** 1 Department of Neurosurgery, Hôpital Erasme, Université Libre de Bruxelles, Route de Lennik 808, 1070 Bruxelles, Belgium; 2 Neurosurgical Service, Beth Israel Deaconess Medical Center, Harvard Medical School, Boston, MA USA; 3 Department of Neurosurgery, The National Hospital for Neurology and Neurosurgery, Queen Square, London, UK; 4 Department of Neurosurgery, Centre Hospitalier de Luxembourg, Luxembourg City, Luxembourg; 5 Department of Neurosurgery, St. Joseph’s University Medical Center, Paterson, NJ USA; 6 Institute of Interdisciplinary Research (IRIBHM), Université Libre de Bruxelles, Brussels, Belgium; 7 Department of Otorhinolaryngology, Hôpital Erasme, Université Libre de Bruxelles, Brussels, Belgium

**Keywords:** Endocrinology, Neurology

## Abstract

Various surgical methods to prevent postoperative cerebrospinal fluid (CSF) leaks during transsphenoidal surgery have been reported. However, comparative studies are scarce. We aimed to compare the efficacy of a fibrin-coated collagen fleece (TachoSil) versus a dural sealant (DuraSeal) to prevent postoperative CSF leakage. We perform a retrospective study comparing two methods of sellar closure during endoscopic endonasal transsphenoidal surgery (EETS) for pituitary adenoma resection: TachoSil patching versus DuraSeal packing. Data concerning diagnosis, reconstruction technique, and surgical outcomes were analyzed. The primary endpoint was postoperative CSF leak rate. We reviewed 198 consecutive patients who underwent 219 EETS for pituitary adenoma from February 2007 and July 2018. Intraoperative CSF leak occurred in 47 cases (21.5%). A total of 33 postoperative CSF leaks were observed (15.1%). A reduction of postoperative CSF leaks in the TachoSil application group compared to the conventional technique using Duraseal was observed (7.7% and 18.2%, respectively; p = 0.062; Pearson exact test) although non-statistically significant. Two patients required lumbar drainage, and no revision repair was necessary to treat postoperative CSF rhinorrhea in Tachosil group. Fibrin-coated collagen fleece patching may be a valuable method to prevent postoperative cerebrospinal fluid (CSF) leaks during EETS for pituitary adenoma resection.

## Introduction

Pituitary adenomas represent approximately 15% of all intracranial neoplasms^1^. Endonasal endoscopic transsphenoidal surgery (EETS) has become the preferred method for treating these tumors^1,2^. While the procedure is considered safe and effective, cerebrospinal fluid (CSF) leak remains a significant complication after EETS^2,3^

Numerous techniques of sellar reconstruction to prevent this complication have been described. Many involve using autologous tissue grafts, including muscle, septal cartilage/bone, fat, or free mucosal flap^1,4–9^. Autologous grafts can lead to additional incisions, increased operative time, risk of additional complications, and patient discomfort^10^. To avoid these limitations, cadaveric acellular dermis or cadaveric fascia lata can be used^7,11,12^. Various adhesive substances that locally reinforce sellar repair can also be used, either alone or in combination, including fibrin sealants or collagen-based compounds^4,13–17^. Finally, vascularized flaps have gained increasing popularity^9,10^. However, the nasoseptal flap (NSF) is not exempt from complications and can lead to nasal discomfort, excessive crusting, anosmia, and the necessity for multiple debridements^9^. The ideal alternative to these techniques would be an option effective at preventing CSF leak, technically simple to handle, inexpensive, and minimizing morbidity^7^.

TachoSil (Takeda Pharma, Wien, Austria) consists of a sheet of collagen, which is coated on one side with human fibrinogen, human thrombin, and riboflavin^4^. The preparation is ready to use and can be applied directly to the target tissue^4^. Recent studies have demonstrated that TachoSil can be useful during EETS, obviating the need for autologous tissue grafts, postoperative lumbar drainage, and NSF reconstruction^2,4,6^.

In this study, we compare the safety and efficacy of a fibrin-coated collagen fleece (TachoSil) patch with fibrin glue versus a dural sealant (DuraSeal, Covidien, Dublin, Ireland) to prevent postoperative CSF leakage.

## Methods

### Patient population

The Erasmus-ULB ethics commitee approved this study (P2018/408) and waived patient consent due to the retrospective study design. Eligible patients were identified from a prospectively maintained institutional pituitary tumor database. All procedures were performed by senior authors (SH and ODW) with extensive experience in pituitary surgery (i.e. with more than 400 transsphenoidal surgeries performed between 1993 and 2007). Eligible patients underwent EETS for pituitary adenoma between February 2007 and July 2018. The primary outcome was postoperative CSF leak rate following sellar repair. Secondary outcomes included the lumbar drain use and reoperation for repeat surgical closure. For each patient, a thorough chart review was conducted, and information obtained included: patient age, gender, tumor size, modified Hardy’s classification for sellar invasion and suprasellar extension, previous surgery, histopathologic diagnosis, intraoperative CSF leakage, repair method, postoperative complications, management of postoperative CSF leak and the length of stay.

### Surgical technique

Under general anesthesia, the patient was placed in a supine position. The approach was performed via one nostril. A 30° rigid endoscope was introduced into the nasal cavity and the middle turbinate was lateralized by gentle pressure with an elevator. The rostrum was partially resected. An anterior sphenoidotomy was performed, and the sphenoid septum was removed. Medtronic electromagnetic neuronavigation system was used to tailor precisely the exposure to the tumor size. After complete tumor removal, the sellar cavity was then explored for evidence of a CSF fistula or tumor remnant. Two different methods were successively used to reconstruct the sella. Accordingly, the patients were divided into cohorts based on the closure technique. In cohort 1 (from 2007 to 2015), we packed the sphenoid sinus with a dural sealant (DuraSeal) alone. In cohort 2 (from 2015 to 2018), we covered the anterior wall of the sella by a single layer of the TachoSil patch and then packed the sphenoid sinus with a fibrin sealant (Tisseel; Baxter Medical, Deerfield, IL, USA).

### Intraoperative CSF leak and repair methods

Intraoperative CSF leaks were classified in operative protocols as « low » flow CSF leaks which are small "weeping" leaks flowing even without Valsalva maneuver and “high” flow CSF leaks which are moderate or large CSF leaks with or without obvious diaphragmatic defect. In the instance of an intraoperative CSF leak, the postoperative cavity dead space was usually filled with a small piece of absorbable gelatin foam (Gelfoam; Upjohn, Kalamazoo, MI) to reduce the amount of CSF leak. For “low” flow CSF leaks, the closure was then performed as described in the surgical technique for each cohort. For “high” flow leaks, an additional covering by an autologous tissue (turbinate cartilage or septal bone) of the sellar opening was used before classical closure for each cohort. Before 2015, we used in our practice a conventional sellar closure technique with the dural sealant closure (DuraSeal). In 2015, as we aimed to reduce the incidence of postoperative CSF leaks, we opted for a promising alternative method using a fibrin-coated collagen fleece (TachoSil).

### Postoperative care

All patients were admitted to the intensive care unit (ICU). On the first postoperative day, patients were transferred to the ward if they were clinically stable and did not present complications.

### Statistical analysis

Continuous variables are summarized by means and standard deviations (SD) and qualitative variables as numbers and percentages. Differences in continuous variables means between two groups were compared using classical Student t-tests or Welch's t-tests in case of variance inequality. Differences in qualitative variables were compared between groups using Pearson's exact chi-square tests. Statistical significance was considered when *p* was < 0.05. All statistical tests were two-sided and performed using IBM-SPSS version 26.0 software (I.B.M. Corp, Armonk, NY, USA) and MedCalc Statistical Software version 14.12.0 (MedCalc Software bvba, Ostend, Belgium).

### Ethical approval

For this type of study informed consent was waived with the acceptance of ULB-Erasmus university hospital’s ethic committee.

### Declaration of helsinki

All methods were carried out in accordance with relevant guidelines and regulations of the 'Declaration of Helsinki'.

## Results

### Patient characteristics

A total of 219 EETS for pituitary adenoma resection were performed on 198 patients at our institution between February 2007 and July 2018. Twenty patients underwent repeat transsphenoidal surgeries for recurrent or residual adenoma. Nineteen patients underwent two operations and one patient three operations. Sellar closure with TachoSil was applied in 65 surgeries, and conventional packing with DuraSeal was applied in the remaining 154 surgeries (Table 1). There were no statistically significant differences between groups for gender, age, and histopathologic diagnoses.

**Table 1:** Demographics variables.

Demographics variables	Tachosil application group	DuraSeal packing group	p value
Number of patients	61	137	
Male (%)	29 (47.5%)	68 (49.6%)	0.878
Mean age, year ± SD	50.7 ± 14.6	50.7 ± 14.6	1.000
**Histopathologic diagnoses**			
Nonfunctioning adenomas	30 (49.2%)	72 (52.6%)	0.760
Functioning adenomas	23 (37.7%)	50 (36.5%)	0.875
Apoplexies	0 (0%)	4 (2.9%)	0.314
Unknown diagnoses (loss of sample, hemorrhage, insufficient sampling)	8 (13.1%)	11 (8.1%)	0.302

Preoperative characteristics of adenomas are shown in Table 2. There were no statistically significant differences between cohorts to the number of macroadenomas, tumor size, modified Hardy's classification for sellar invasion, and the number of previous surgeries. However, the suprasellar extension was significantly more likely in the TachoSil treatment group when compared to the conventional packing group (40% versus 25.3%; p = 0.036) based on the modified Hardy's system.

**Table 2:** Preoperative characteristics of adenomas.

Preoperative variable	Tachosil application group	DuraSeal packing group	p value
Number of surgery	65	154	
Macroadenoma	56 (86.2%)	129 (83.8%)	0.690
Mean size, mm ± SD	20.9 ± 8.5	21.9 ± 10.6	0.441
**Modified Hardy’s classification**			
I	9 (13.8%)	25 (16.2%)	0.690
II	52 (80%)	104 (67.5%)	0.730
III	1 (1.5%)	12 (7.8%)	0.115
IV	3 (4.6%)	13 (8.4%)	0.404
**Modified Hardy’s system**			
0	12 (18.5%)	54 (35.1%)	0.016
A	26 (40%)	39 (25.3%)	0.036
B	14 (21.5%)	28 (18.2%)	0.577
C	13 (20%)	33 (21.4%)	0.858
Previous surgery	12 (18.5%)	41 (26.6%)	0.153

Complications related to EETS according to the closure method are shown in Table 3. There was no statistically significant difference between the TachoSil application group and conventional technique to the amount of "high" flow intraoperative CSF leaks (16.7% versus 40%, respectively; p = 0.176). A non-statistically significant reduction of postoperative CSF leaks in the TachoSil application group compared to conventional technique using Duraseal was observed (7.7% and 18.2%, respectively; p = 0.062; Pearson exact test). Cohorts were not significantly different when secondary outcomes were analyzed. Specifically, there were no differences in the use of Diamox (7.7% and 16.2%, respectively; p = 0.131), lumbar drainage (3.1% and 7.1%, respectively; p = 0.353) or surgical revision repair (0% and 2.6%, respectively; p = 0.321). The length of hospital stay was also not significantly different between the groups; p = 0.280. Two patients died in the intensive care unit postoperatively. The first is due to an unrelated aneurysm rupture, the second following iatrogenic vascular injury.

**Table 3:** Complications related with transsphenoidal approach surgery according to closure method.

Surgical variable	Tachosil application group	DuraSeal packing group	
**Intraoperative CSF leak**	12/65 (18.5%)	35/154 (22.7%)	p = 0.590
Low flow	10/12 (83.3%)	21/35 (60%)	
High flow	2/12 (16.7%)	14/35 (40%)	p = 0.176
Postoperative CSF rhinorrhea	5/65 (7.7%)	28/154 (18.2%)	p = 0.062
Diamox	5/65 (7.7%)	25/154 (16.2%)	p = 0.131
Postoperative lumbar drainage	2/65 (3.1%)	11/154 (7.1%)	p = 0.353
Revision repair	0/65 (0%)	4/154 (2.6%)	p = 0.321
Meningitis	1/65 (1.5%)	4/154 (2.6%)	p = 1.000
Death	0/65 (0%)	2/154 (1.3%)	p = 1.000
Mean length of hospital stay, days ± SD	7.1 ± 4.1	7.8 ± 4.6	p = 0.280

*CSF* cerebrospinal fluid.

## Discussion

EETS has become the preferred method for the treatment of many pituitary adenomas^1,2^. However, the approach has limitations; chief among them is the risk of CSF leak^2,3,14^. In our study, we compared two types of sellar closure techniques following EETS. A decreased postoperative CSF leaks occurrence rate in the TachoSil group compared to Duraseal group was observed but did not reach the prespecified significance level. Along these lines, the Tachosil cohort required lumbar drainage in 2 cases (3.1% versus 7.1%), and no patient required revision repair surgery (0% versus 2.6%) without significance.

Interestingly, there were significantly more patients with suprasellar extension in the Tachosil cohort, a known predictor of postoperative CSF leaks^1^. In this context, our findings are more striking and may suggest that Tachosil closure has greater efficacy at preventing CSF leak than Duraseal packing alone. Other predictors of postoperative CSF leak, such as intraoperative CSF leak, tumor size, and repeat surgery, were not significantly different between the cohorts^1,2^.

TachoSil has several potential advantages for sellar reconstruction when compared to other techniques. First, it serves as a reliable barrier by attaching firmly to dura. Its placement can be performed in a narrow field with straightforward surgical handling. From our experience, it can be easily removed in recurrent tumor surgery. Finally, it provides hemostasis with antigenicity—a feature associated with a lower incidence of postoperative infection^2^.

While our study found fewer postoperative CSF leaks in the Tachosil cohort, it remains higher than other studies using the same material for sellar closure (Table 4). A possible explanation is a difference in technique. Hong et al. used Tachosil as part of a "sandwich technique" in 101 patients during EETS, and only two (1.9%) developed postoperative CSF rhinorrhea^4^. In another study performed on 19 grade 3 intraoperative CSF leaks, postoperative CSF leakage following Tachosil repair utilizing the "sandwich technique" was 5.3%^2^. Tamasauskas et al. performed a sellar closure using Tachosil and Surgicel (Ethicon, NJ, USA) in 29 patients who underwent EETS, and none had a postoperative CSF leak^6^. Currently, the "sandwich technique" using Tachosil appears more effective at preventing postoperative CSF leaks than a single layer technique, which was utilized in our study.

**Table 4:** Summary of studies using Tachosil for sellar closure during transsphenoidal surgery.

Closure technique	Approach	Material	N patient exposed	Intraoperative CSF leak	PO CSF leak	LD	Surgery	Remark
TACHOSIL	EETS (present sudy)	One layer Tachosil + Tissucol	65	12	7.7% (5/65)	2	0	Cost Virus transmission + easily removed in revision repair + immunologically well tolerated + hemostasis + lower postoperative infection
	MTS ± endoscope^15^	Sandwich technique	101	18	1.9% (2/101)	0	0	
	MTS^20^	Sandwich technique	19	19	5.3% (1/19)	0	0	
	MTS − EETS^21^	Surgicel + Tachosil	29	29	0% (0/29)	0	0	

*N* number, *PO* postoperative, *LD* lumbar drainage, *EETS* endonasal endoscopic transphenoidal surgery, *MTS* microscopic transsphenoidal surgery.

Numerous other techniques of sellar floor reconstruction have been described, and all have potential benefits and limitations^5^. Classically, surgeons have used autologous materials such as abdominal fat, muscle, or free mucosal graft with or without support for the graft from nasal bone or cartilage to prevent postoperative CSF rhinorrhea^1,4–9^. These methods are effective for this purpose, with a rate of postoperative CSF leaks ranging from 0 to 10%, similar to our study^1,4–9,17^. While these grafts have the advantage of compatibility and cost, harvesting them can prolong the operative time and often requires a separate surgical incision, which can also be associated with complications (wound dehiscence, infection, scarring and hematoma) and cause additional discomfort to patients^7^. Fat may also interfere with the interpretation of the sellar content on postoperative MRI^3^. Inadequate packing may aggravate the arachnoid tearing and compress the optic chiasm^5^. These limitations are partially addressed by using cadaveric materials (acellular dermis or cadaveric fascia lata^7,11,12^. A postoperative CSF leak rate of 5.5 and 7.6% was demonstrated in two retrospective studies using AlloDerm (LifeCell Corporation, Woodlands, TX) for sellar floor reconstruction in transsphenoidal surgery (Table 2)^7,11^. However, no postoperative CSF leak was encountered in the study performed by Fiorindi et al. using a cadaveric fascia lata for sellar closure^12^. These grafts also have limitations and may not encourage healing as autologous grafts and may cause MRI interference^5,10^.

Vascularized flaps are currently thought to be the most effective technique for endoscopic endonasal reconstruction^9,10^. In a systematic review of 38 studies by Harvey et al., 609 patients with significant dural defects were identified^18^. From this cohort, 326 underwent free graft reconstruction while 283 underwent vascularized reconstruction, resulting in a significantly different postoperative CSF leak rate of 15.6% (51 of 326) and 6.7% (19 of 283), respectively^18^. In a series of 151 patients with intraoperative CSF leaks of whom 144 received Hadad-Bassagestaguy nasoseptal flaps, only 3.3% developed postoperative CSF leak (Table 2)^19^. Another retrospective study using NSF in thirty-one grade III CSF leaks demonstrated a persistent postoperative CSF leak rate of 6.4% (Table 2)^1^. Barger et al. have developed a minimal posterior NSF technique with a postoperative CSF leak rate of only 2.3% (Table 2)^10^. This type of vascularized flap does not seem to prolong the duration of surgery or generate postoperative nasal complications as much as larger flaps^10^. Due to the limitations of the techniques mentioned above, many authors have attempted to obviate autologous and cadaveric tissue grafts. Indeed, fibrin sealants have been used for sellar floor closure with comparable postoperative CSF leakage rates of between 0 and 12.5% and appear to be effective with an acceptable safety profile^13–17,20^. However, they have limitations, including the possibility of viral transmission and ethical concerns from patients, as these are derived from animals^5,17^. DuraSeal is entirely synthetic and is reabsorbed^17^. Thus, unlike fibrin sealants, the potential for viral transmission is eliminated^3^. Pereira et al. have demonstrated a postoperative CSF leakage rate of only 5.6% with the use of DuraSeal in 180 sellar closures during EETS (Table 2)^17^. Our study has shown a postoperative CSF leak rate of 18.2% using DuraSeal alone, with 11 patients requiring lumbar drainage and four repairs surgery. However, unlike our study where Duraseal alone was used for packing, Pereira et al. utilized Duraseal in combinations with fat, Spongostan (Ethicon, NJ, USA), and Floseal (Baxter Inc, IL, USA). Nevertheless, our results suggest a significantly higher rate of postoperative CSF leak when using Duraseal alone.

Some authors have advocated a sellar reconstruction algorithm using a combination of methods, depending on the significance of the CSF leak^17^. For example, Zhou et al. have demonstrated that only 6 of the 492 (1.2%) cases using a graded repair method subsequently developed postoperative CSF leak (Table 5)^1^. Jalessi et al. analyzing 240 cases, reported a postoperative CSF leak rate of 0.8%, despite 44% of cases presenting intraoperative CSF leak (Table 5)^22^. Similarly, Esposito et al., has a postoperative CSF leak rate of 2.5% utilizing a graded repair method (Table 5)^21^. In these studies, lumbar drainage was placed at the end of surgery if a high output intraoperative CSF leak was identified^21,22^. While lumbar drains reduce intracranial pressure and may hasten the healing of the sellar floor, they may also be associated with severe complications^5,17^. In our study the Tachosil cohort only required two lumbar drainages, compared to 11 in the Duraseal cohort.

**Table 5:** Summary of various techniques for sellar closure during transsphenoidal surgery.

Closure technique	Approach	Material	N patient exposed	Intraoperative CSF leak	PO CSF leak	LD	Surgery	Remark
Free autologous graft	MTS^8^	Muscle + septal cartilage	23	23	0% (0/23)	0	0	+ Totally compatible + Free of charge − ↑ operative time − Separate incision (complications) − Additional discomfort − MRI interference − Inadequate packing: optic chiasm compression, ↑ Arachnoid tearing
	MTS − EETS^21^	Fat + autologous bone	29	29	10% (3/29)	2	1 repair	
	EETS^23^	Fat graft + artificial dura	55	55	7.3% (4/55)	4	2 repairs	
	EETS − METS ± endoscope^15^	Fat graft + fibrin glue	54	15	9.3% (5/54)	22	–	
	MSTS − EETS − hybrid^12^	Fat	87	7	9.2% (8/87)	–	2 repairs	
	EETS^19^	Fat + autologous bone/cartilage + glue	235	–	1.7% (4/235)	4	1 repair + LD	
	EETS^18^	Collagen dural graft + nasal cavity floor free mucosal graft + oxidized cellulose + polyethylene glue + Bioresorbable packing	50	20	0 (0/50)	0	0	
Cadaveric graft	ETTS^11^	Fat + cadaveric fascia lata + Fibrin glue	16	16	0% (0/16)	9	0	+ No separate incision − MRI interference − Not support healing as living tissue
	MSTS − EETS − hybrid^12^	Alloderm	163	8	5.5% (9/163)	–	2 repairs	
	MTS − EETS^7^	Alloderm + cartilage/bone autograft + fibrin glue	13	5	7.6 (1/13)	1	0	
Pediculized flap	3 EETS^1,22,23^	Various pediculized flap (144 NSF)	151	151	3.3% (5/151)			+ Rapid/effective integration − ↑ operative time − ↑ healing period − Nasal complaints (crusting)
		Fascia graft + fat graft + NSF	31	31	6.4% (2/31)	2	1 repair	
		Posterior NSF (If leak: Alloderm + NSF ± fat graft ± LD)	43	21	2.3% (1/43)	2	1 repair + LD	+ Small flap − Limitation of covered areas − LD complications
Fibrin sealant	5 EETS^3–5,14,17^	Gelatin sponge	28	28	3.6% (1/28)	0	1 VPS	+ Reabsorbed − Virus transmission -bovine spongiform encephalitis − animal derivatives against patient wishes
		Collagen fleece	29	29	6.9% (2/29)	6	0	
		–	40	40	0% (0/40)	0	0	
		Collagen foil	15	9	6.7% (1/15)	0	1 repair	
		Different combinations: fat − spongostan − floseal	16	–	12.5% (2/16)	–	–	
Duraseal	EETS^17^	Different combinations: fat − spongostan − floseal	180	–	5.6% (10/180)	–	–	+ Synthetic: no disease transmission + Immunologically well tolerated + Reabsorbed
Graded repair method	EETS^16^	Stage I: Surgicel + Gelfoam Stage II: fat + fascia + same as Stage I Stage III: same as Stage II + surgical glue ± LD	240	107	0.8%	1	1	LD complications
	EETS^10^	Grade 0: collagen sponge Grade 1: collagen sponge + titanium mesh buttress Grade 2: fat grafts + same as grade 1 Grade 3: same as Grade 2 + LD	668	380	2.5% (17/668)	6	11	LD complications

*N* number, *PO* postoperative, *LD* lumbar drainage, *MTS* microscopic transsphenoidal surgery, *EETS* endonasal endoscopic transphenoidal surgery, *NSF* nasoseptal flap, *VPS* ventriculoperitoneal shunt, + advantage, −  disavantages.

In a laboratory study, Chauvet et al. found the mean pressure at which a leak visually occurred with Bioglue (CryoLife, Inc, GA, USA), Duraseal, Tachosil and Tissucol (Baxter Healthcare, IL, USA) was 16.78, 28.31, 27.09 and 10.03 mmHg, respectively, which suggest that Duraseal and Tachosil may be superior for closure^23^. Two types of leaks were reported: those occurring between the sealant and the dura (Bioglue, Duraseal and Tissucol) and those occurring through the sealant (Tachosil)^23^. These results could explain why "the sandwich technique," using two layers of Tachosil, seems superior to the single-layer technique, as it prevents the second time of the trans-graft leak.

Besides its retrospective design, the main limitation of this study is the sequential use of the two different techniques investigated (i.e. Duraseal then Tachosil). One could expect that experience gained during the first part of the study (i.e. Duraseal) may lead to bias in favor of the last technique used (i.e. Tachosil). However, this potential bias is limited by the extensive prior experience of the operators—although mainly microscope-assisted pituitary surgeries.

## Conclusion

Fibrin-coated collagen fleece patching may be a valuable method to prevent CSF leaks during EETS for pituitary adenoma resection. This study reports fewer postoperative leaks in the TachoSil cohort compared to the Duraseal cohort without reaching significance. This observation dovetails with previous surgical series and experimental data but powered studies to achieve higher levels of evidence are required to confirm these results.
